# Long non-coding RNA GHET1/miR-105/RAP2B axis regulates the progression of acute myeloid leukemia

**DOI:** 10.7150/jca.47294

**Published:** 2020-10-17

**Authors:** Yue Xiao, Tangfei Li, Qianfu Xue, Lili Miao

**Affiliations:** Yongchuan Hospital of Chongqing Medical University, Yongchuan, Chongqing, China.

**Keywords:** lncRNA GHET1, acute myeloid leukemia, miR-105, *RAP2B*

## Abstract

**Background:** To explore the biological effects and potential molecular mechanisms of long non-coding RNA (lncRNA) gastric carcinoma proliferation enhancing transcript 1 (GHET1) in acute myeloid leukemia (AML).

**Methods:** Fluorescence *in situ* hybridization was performed to determine the location of GHET1. Quantitative polymerase chain reaction (qPCR) was performed to verify RNA expression. GHET1 overexpression and knockdown were achieved by transfection of the expression vector or short hairpin RNA. Western blotting, qPCR, Cell Counting Kit-8 assay, JC-1 staining, and flow cytometry were performed to measure GHET1 function. The dual luciferase reporter assay was performed to confirm the relationship between microRNA 105 (mir-105) and Ras-related protein Rap-2B (*RAP2B*).

**Results:** GHET1 was localized in the nucleus of NB4 cell lines. GHET1 expression was elevated in AML cell lines compared with normal bone marrow mononuclear cells. GHET1 knockdown led to inhibition of proliferation and promoted the differentiation and apoptosis of AML cell lines. Furthermore, GHET1 directly bound to miR-105 and downregulated miR-105 expression. MiR-105 overexpression suppressed proliferation and induced the differentiation and apoptosis of AML cell lines. In addition,* RAP2B* was confirmed to be a target gene of miR-105 and an inverse correlation was shown between their expression levels in AML cell lines; when miR-105 increased, Rap-2B level decreased and vice versa*.*

**Conclusion:** This study demonstrated that the GHET1/miR-105/*Rap2B* axis may be a critical signaling pathway involved in AML progression.

## Introduction

The most common acute leukemia is acute myeloid leukemia (AML) in adults. It is a malignant clonal disease that originates from myeloid hematopoietic stem/progenitor cells. It is characterized by uncontrolled proliferation and blocked apoptosis of immature myeloid progenitor cells, and its molecular mechanism and treatment results are highly heterogeneous 1. Genetic abnormalities and epigenetic changes are important links in the pathogenesis of AML 2. In recent years, several studies have shown that a variety of long non-coding RNAs (lncRNAs) are significantly correlated with the occurrence and development of leukemia 3-6.

LncRNA is a functional RNA molecule located in eukaryotic cells, which is generally longer than 200 nucleotides in length and is located in the nucleus or cytoplasm of eukaryotic cells. It has little or no protein-coding function, and regulates gene expression at the epigenetic, transcriptional, and post-transcriptional levels, mainly by interacting with proteins, DNA, or RNA 7.

LncRNA plays an important role in the proliferation, cloning, apoptosis, invasion, metastasis, and drug resistance of various types of tumor cells (e.g., liver cancer, prostate cancer, lung cancer, melanoma). LncRNA gastric carcinoma proliferation enhancing transcript 1 (GHET1) is located on chromosome 7q36.1, and is 1913 nucleotides in length 8. Studies have shown that lncRNA GHET1 is highly expressed in gastric, bladder, and colon cancers; promotes the malignant progression of tumors; and is associated with tumor size, invasive capacity, and poor prognosis 9-11. Bioinformatics analysis showed that lncRNA GHET1 can potentially target miR-105; Ras-related protein Rap-2B (*RAP2B*) is the target gene of miR-105 and plays a role in cell proliferation and differentiation. As a novel miRNA regulator, the biological function of miR-105 has been confirmed in a variety of malignant diseases. Studies have shown that miR-105 expression can be upregulated in breast cancer (including triple-negative breast cancer) 12, 13 and colorectal cancer 14, functioning as a tumor promoter that targets a variety of genes. Additionally, some studies have shown that miR-105 expression can be downregulated in glioblastoma 15 and hepatocellular carcinoma 16, 17. MiR-105 acts as a tumor suppressor or an oncogene, depending on the particular tumor context and base-pairing genes. The *RAP2B* gene belongs to the *RAS* oncogene superfamily. Current research on *RAP2B* shows that it mainly plays a role as a cancer-promoting factor in a variety of diseases, and plays an important role in the migration and invasion of tumor cells such as lung cancer 18, renal cell carcinoma 19, and prostate cancer 20.

The role of lncRNA GHET1 in AML has not been investigated, and it remains unclear whether the significant roles of GHET1 in AML are correlated with the dysregulation of miR-105. This paper will explore the expression of GHET1 in AML and its possible biological effects, and determine its possible molecular mechanisms in AML tumorigenesis.

## Methods

### Ethics approval

This study was approved by the Ethics Committee of Yongchuan hospital of Chongqing Medical University (Chongqing, China) and all the protocols conformed to the Ethical Guidelines of the World Medical Association Declaration of Helsinki. Before participating in the study, informed consent was obtained from all participating individuals.

### Human leukemia cell lines

Human leukemia cell lines NB4 and HL-60 were purchased from ATCC (American Type Culture Collection, Manassas, VA, USA). Cells were cultured in RPMI-1640 media supplemented with 15% fetal bovine serum (FBS), 100 U/mL penicillin, 100 mg/mL streptomycin, and 2 mM L-glutamine (Gibco, Life Technologies, Carlsbad, CA, USA). HEK293T cells were cultured in Dulbecco's Modified Eagle Medium-high glucose media with 10% FBS, 100 U/mL penicillin, 100 mg/mL streptomycin, and 2 mM L-glutamine. These cells were cultured at 37 °C in a humidified incubator with 5% CO_2_ (Gibco, Life Technologies, Carlsbad, CA, USA).

### Transfection, virus package, and infection

The lncRNA GHET1 expression vector was constructed by GenChem (Shanghai, China), and the miR-105 mimic and inhibitor were synthesized by Ribo Life Science (Soochow, China). Transient transfections in HEK293T cells were performed using polyethyleneimine (PEI) (Polysciences, Warrington, PA, USA) in the OPTI-MEM medium (Life Technologies, Carlsbad, CA, USA) with a ratio of 1:4 to 1:6 of DNA:PEI. The transfection of human leukemia cells was achieved by an electroporation device system according to the manufacturer's instructions (Neon Transfection System; Invitrogen, Basel, Switzerland). For transfection, 140,000 cells were taken up in 10 μL resuspension buffer containing 0.8 μg of the plasmid with 10 μL Neon tips using a Neon Transfection System pipette. We used the following parameters for the NB4 and HL-60 cells: pulse voltage (1400 V), pulse width (20 ms), and pulse number (1 time). Cells were changed to complete media 6 h after transfection, and collected 48 h post-transfection for further designed experiments. Viral particles were produced by HEK293T cells in a 10 cm dish transfected with 4 μg PMD2G and 6 μg PSPAX2 packaging plasmids (Addgene, Watertown, MA, USA), together with 8 μg lentiviral expression vectors encoding target genes including pCMV-GFP, pCMV-GHET1-GFP, or pSUPER vector encoding short hairpin RNAs (shRNAs). Supernatant carrying the viral particles was harvested 35 and 60 h after transfection and concentrated to 100 × volume by Poly (ethylene glycol) 8,000 (Sigma-Aldrich, St. Louis, MS, USA). For viral infection, 1×10^6^ human leukemia cells were seeded in 1 mL new complete media for 6 h, followed by the addition of 50 μL viral supernatant and 8 μg/mL polybrene, and centrifugation at 1800 rpm for 45 min at 20 ºC. At 12 h after spinfection, the medium was changed and cells were cultured for another 48 h.

### Fluorescence *in situ* hybridization assay

Amine-modified RNA was synthesized and labeled with fluorescent dye. Before fluorescence *in situ* hybridization (FISH), the sample contact area was covered with 500 µL 10% neutral buffered formalin, and the cells were fixed at 25 °C for 30 min. After fixation, the formalin was discarded and the cells were washed one time in 1× phosphate-buffered saline (PBS). Then samples were dehydrated in ethanol (50%, 80%, and 95% (v/v) series, incubated with 300 µL ethanol for 3 min at each concentration) before hybridization. Using a wet-sealed slide incubation chamber, the samples were hybridized at 55 °C for 15 min. Briefly speaking, 500 µL volumes of hybridization buffer (0.7 M NaCl, 0.1 M Tris [pH 8.0], 0.1% sodium dodecyl sulfate, 10 mM EDTA, containing probe, preheated to 55 °C) were applied to the surface of the tape and the lid of chamber was sealed, creating a humid, temperature-controlled environment inside the chamber. After 15 min, the samples were briefly rinsed with probe-free hybridization buffer. Then, the samples were preheated to 55 °C. The cells were counterstained for 10 min in the dark with ~30 µL mounting medium containing 1.5 µg ml-1 4',6-diamidino-2-phenylindole (DAPI). Then, the cells were mounted with a cover glass and examined by fluorescence microscopy.

### Cell proliferation assay

Three independent experiments were performed for the cell proliferation assay. Human leukemia cells (5×10^3^) were treated in 96-well plates for the indicated times and then 10 μL Cell Counting Kit-8 (CCK-8) (Dojindo Molecular Technologies, Inc., Rockville, MD, USA) was added to 100 μL culture media. Cells were incubated at 37 °C for 1-4 h, and then the plates were read at 450 nm by a microplate reader (Model 550, Bio-Rad Laboratories, Richmond, CA, USA). The following formula was used to calculate cell viability (%) = OD value of treatment group/OD value of control group 100×.

### RNA extraction and quantitative PCR

Total RNA was extracted by using Trizol (Life Technologies, South San Francisco, CA USA), and total RNA (2 μg) was reverse transcribed into cDNA by using the 5× All-In-One reverse transcription MasterMix (ABM, Vancouver, Canada). Quantitative PCR (qPCR) was performed by mixing the cDNA, gene-specific primers, and EvaGreen 2× qPCR MasterMix (ABM) in the QuantStudio 3 Real-Time PCR System (Applied Biosystems, Foster City, CA, USA). Primer sequences were:

- GHET1-F: CGCAAGGTACCAGAGAGCCG;
- GHET1-R: AGTGGCGCTTGo CTGGGATTT;
- ITGAM-F: GCTTTGGTGGCTTCCTTGTG;
- ITGAM-R: TAGTCGCACTGGTAGAGGCT;
- miR-105-F: GTGCATCGTGGTCAAATGCT;
- miR-105-R: ACACCGTAGCACATGCTCAA
- *RAP2B*-F: AAGCCTCGGTAGACGAGCTA;
- *RAP2B*-R: GTCGGATGCGTTTGGCTTTT;
- GAPDH-F: CTGGGCTACACTGAGCACC;
- GAPDH-R: AAGTGGTCGTTGAGGGCAATG.

### shRNA design

Primers were designed against sequences 5′-GCTATCTCTGGTCAAGTCA-3′, 5′-GCTTCCAGCGTCGGTAAAT-3′ and 5′-GGTCATAATCGAAGTATCT-3′ for human GHET1 and cloned in the pSUPER (Oligoengine, Seattle, WA, USA). Then, a KpnI-XbaI fragment containing the H1 promoter for RNA polymerase III and the shRNA sequence were excised from pSUPER and subcloned into the pLVTHM plasmid (#12247; Addgene, Watertown, MA, USA).

### Flow cytometry analysis

Human leukemia cells were cultured in 12-well plates and treated with or without the vGHET1 expression vector or shRNA, and with or without miRNA-105 mimic or miRNA-105 inhibitor. After culturing at 37 ºC for the indicated times, the apoptosis assay was performed using the Annexin V-FITC Apoptosis Detection Kit (Sigma-Aldrich, St. Louis, MO, USA). Then, 1×10^5^ cells were stained in the dark with 5 μL Annexin V-FITC and 1 μL propidium iodide (PI). These cells were analyzed with FACS Calibur. The flow cytometry data were analyzed using CellQuest 3.0 software (BD Biosciences, Franklin Lakes, NJ, USA). Cells negative for Annexin V and PI staining were considered viable cells, cells positive for Annexin V staining and negative for PI staining were considered early apoptotic cells, and cells positive for Annexin V and PI staining were considered late apoptotic cells. Following the specified protocol, the harvested cells were washed twice with cold phosphate-buffered saline (PBS), resuspended in 50 μL PBS, and cultured for 30 min with phycoerythrin (PE)-conjugated anti-CD11b (BD Biosciences, San Jose, CA, USA) in the dark. Cells were washed with PBS twice and resuspended in 200 μL PBS. The percentage of CD11b-positive cells was measured by the Accuri C6 flow cytometer (BD Biosciences, CA, USA).

### JC-1 staining

Loss of mitochondrial membrane potential of human leukemia cells was measured by the MitoProbe assay (Molecular Probes, Invitrogen, Eugene, OR, USA). Briefly, GBT (50 nM) was used to treat human leukemia cells for 24 h, followed by incubation with JC-1 (5 µM) for 30 min in the dark at 37 °C. After being washed with PBS three times, cells were analyzed immediately using the Zeiss 4.4.0 Axiovert 200 Inverted Fluorescence Microscope with a 100 W mercury lamp under the following conditions: 330-385 nm excitation filter (excf), 400 nm dichroic mirror (dm), and 420 nm barrier filter (bf) for Hoechst 33258; 450-480 nm excf, 500 nm dm, and 515 nm bf for JC-1.

### Luciferase assay

For the luciferase assay, *RAP2B*-3′UTR-wild-type and mutant segments were amplified by PCR and cloned into the pGL3 luciferase reporter control vector. Either wild-type or mutant vector was co-transfected along with miRNA-105 mimic or scramble into HEK293T cells using polyethyleneimine (PEI) (Polysciences, Warrington, PA, USA) in OPTI-MEM medium (Life Technologies, Carlsbad, CA, USA) at a DNA:PEI ratio of 1:4 to 1:6. At 48 h after transfection, luciferase activity was detected using the dual-luciferase reporter assay system (Promega, Madison, WI, USA), and the signal was captured using the SpectraMax M5 multi-detection microplate reader (Molecular Devices Corporation, Sunnyvale, CA, USA).

### Western blotting

Protein lysates were prepared in RIPA buffer (50 mM Tris-Hcl pH 7.5, 150 mM NaCl, 10 mM EDTA, 0.5% sodium deoxycholate, 1% NP-40, 1 mM sodium ovanadate, 10 μg/mL aprotinin, 1 mM phenylmethanesulfonyl fluoride, and 10 μg/mL leupeptin) supplemented with complete protease inhibitors (Roche, Indianapolis, IN, USA). The protein concentration was tested using the BCA protein assay kit (Thermo Fisher Scientific, Carlsbad, CA, USA). Cell lysate (50 μg) was separated by sodium dodecyl sulfate polyacrylamide gel electrophoresis and transferred to nitrocellulose membranes (Pall Corporation, Washington, NY, USA). Membranes were blocked in 5% non‑fat milk for 1 h at room temperature and probed overnight at 4 ºC with specific antibodies: anti-rabbit RAP2B, cleaved poly (ADP ribose) polymerase (c-PARP), PARP, or anti-mouse β-actin (Cell Signaling Technology, Danvers, MA, USA). Membranes were washed three times with PBS with Tween detergent (PBST) the next day, and then incubated with horseradish peroxidase-conjugated secondary antibodies for 1 h at room temperature. Finally, the membranes were washed again three times with PBST, and proteins were visualized using an enhanced chemiluminescence system (Millipore, Los Angeles, CA USA). The representative western blot images for at least three independent experiments have been cropped and auto-contrasted.

### Statistical analysis

The statistical details of experiments can be found in the figure legends. For at least three independent experiments, all data are shown as the mean ± standard deviation. The paired two-tailed Student's *t*-test or Mann-Whitney nonparametric test was used for *in vitro* experiments, and two-way analysis of variance plus the Bonferroni post hoc test was used for *in vivo* experiments. *P* ≤ 0.05 was considered statistically significant. *, *p* ≤ 0.05; **,* p* ≤ 0.01, compared with the controls, respectively.

## Results

### GHET1 is localized in the nucleus of NB4 cell lines

**Figure 1:**
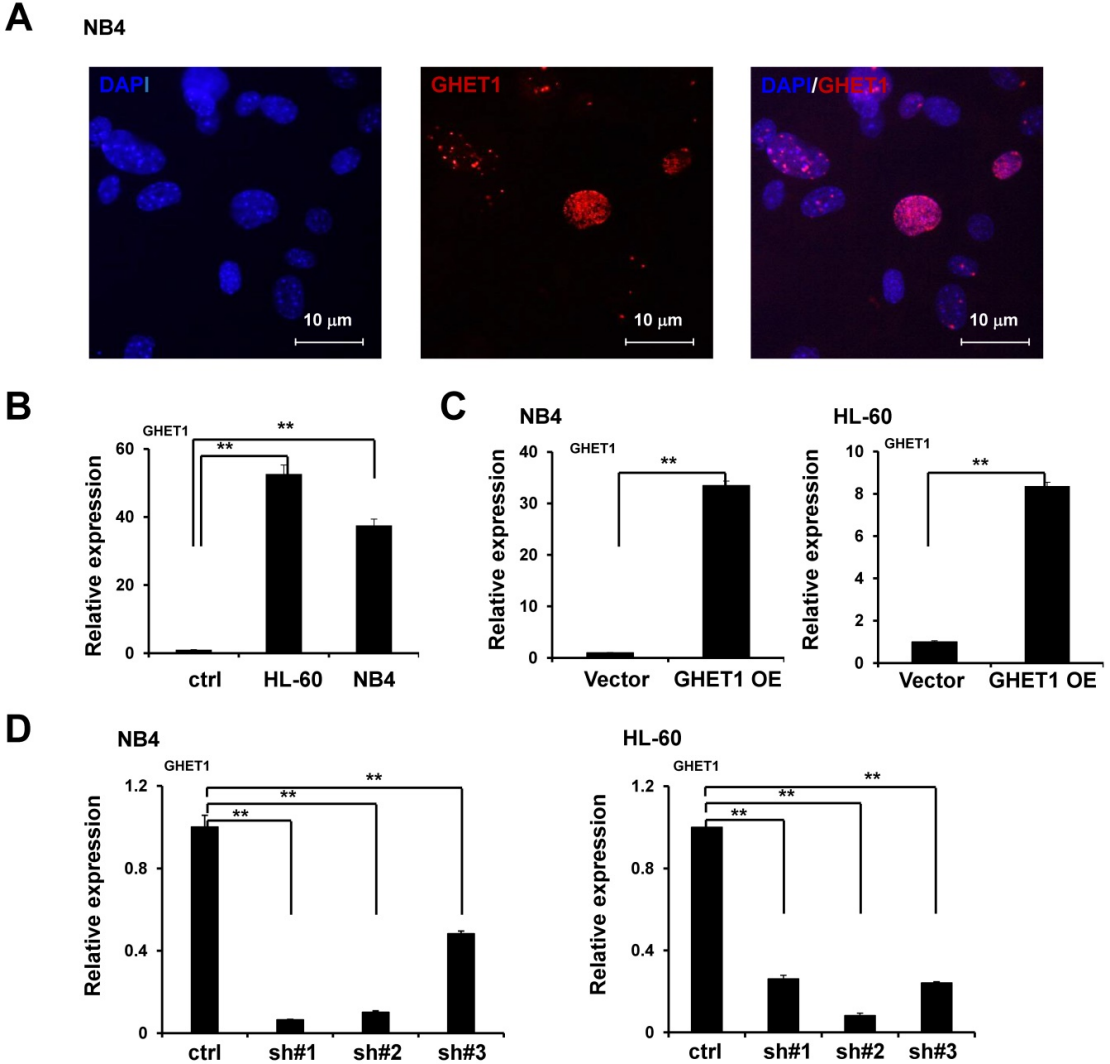
** Subcellular localization and expression of GHET1 in AML cell lines.** (**A**) FISH demonstrated that GHET1 is localized in the nucleus of AML cells. (**B**) GHET1 was upregulated in AML cells compared with normal bone marrow mononuclear cells by qPCR analysis. (**C**) GHET1 was significantly upregulated in AML cells after infection by lentivirus carrying lncRNA GHET1 expressing vector compared with that after infection of vector only. (**D**) GHET1 was significantly downregulated after NB4 and HL-60 cell lines were infected by GHET1 shRNA, respectively. *, p ≤ 0.05, **, p ≤ 0.01.

We performed RNA-FISH to detect GHET1 subcellular localization in AML cell lines. We found that GHET1 was mainly distributed in the nucleus of NB4 cell lines (**Fig. 1A**)**.** To test the expression of GHET1, the qPCR assay was performed to detect its expression in normal bone marrow mononuclear cells and AML cell lines, including NB4 and HL-60 cell lines. Our results showed that GHET1 expression was clearly increased in AML cell lines compared with normal bone marrow (**Fig. 1B**). These data confirm the elevation of GHET1 in AML, suggesting that GHET1 may play a vital role in the incidence of AML. To explore the biological function of GHET1 in AML cells, we infected NB4 and HL-60 cell lines with lentivirus carrying lncRNA GHET1 expressing vector to overexpress GHET1. The infection efficiency was detected by qPCR. The expression of GHET1 was significantly increased after infection of lentivirus carrying lncRNA GHET1 expressing vector in AML cell lines compared with infection of vector only (**Fig. 1C**). Furthermore, we infected NB4 and HL60 cell lines with lentivirus carrying lncRNA GHET1 shRNAs (3 shRNA) to knockdown the expression of GHET1, and the infection efficiency was detected by qPCR. These results showed that GHET1 had the lowest expression after the infection of shRNA#1 in NB4 cell lines, and the lowest expression after the infection of shRNA#2 in HL60 cell lines (**Fig. 1D**). In subsequent experiments, endogenous GHET1 was knocked down by shRNA#1 and shRNA#2 in NB4 and HL60 cell lines.

### GHET1 plays a role in the proliferation, differentiation, and apoptosis of AML cell lines

**Figure 2:**
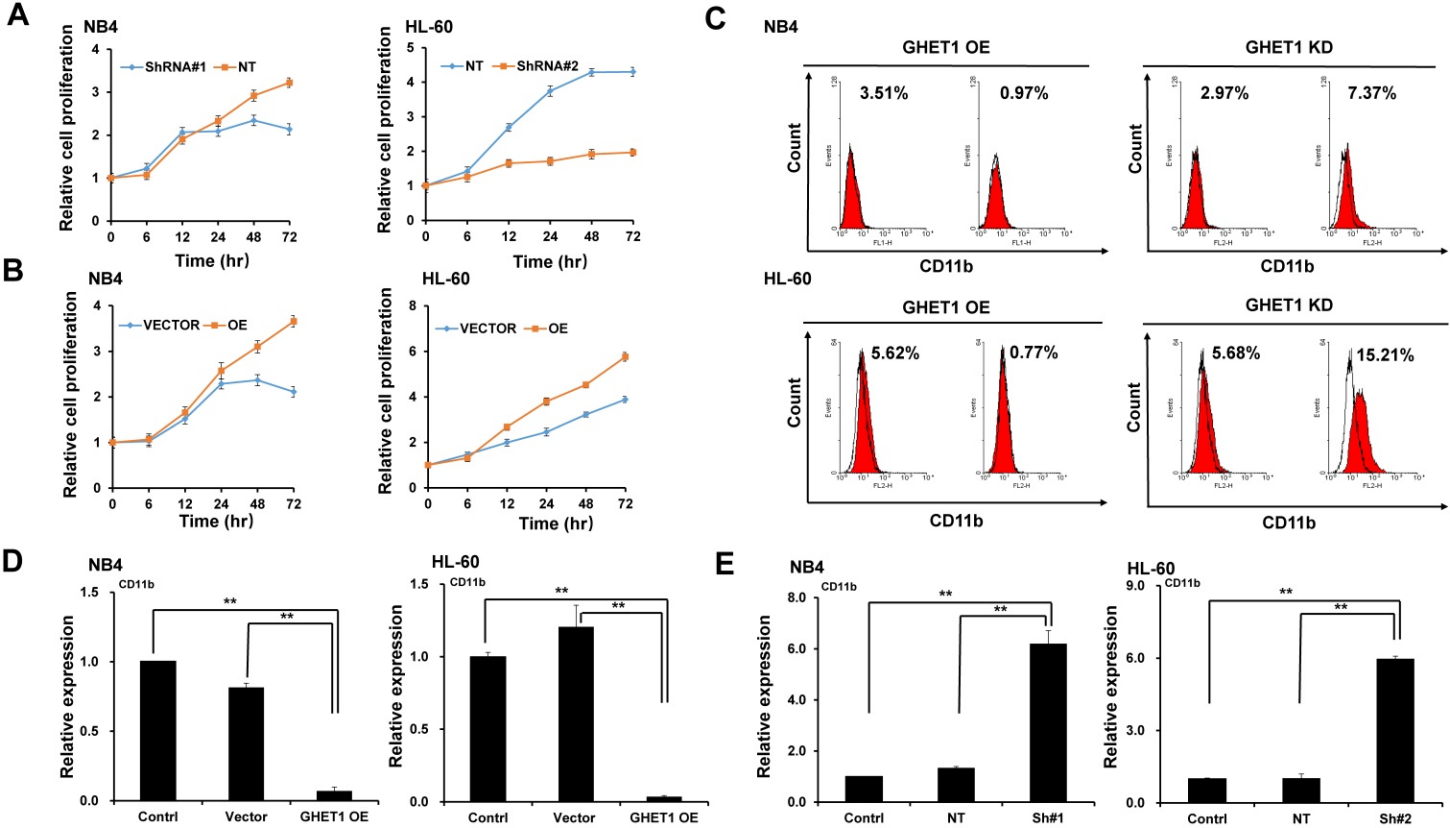
** Effects of GHET1 on cell proliferation and differentiation.** Cell proliferation assay of NB4 and HL60 cells was performed by transfecting with GHET1 overexpression vector or GHET1 shRNA by the CCK-8 assay. (**A**) GHET1 knockdown inhibited cell proliferation, (**B**) and GHET1 overexpression promoted cell proliferation. The CD11b expression in NB4 and HL60 cells with GHET1 overexpression or knockdown was detected by (**C**) flow cytometry and (**D, E**) qPCR. GHET1 overexpression inhibited cell differentiation, and GHET1 knockdown promoted cell differentiation. *, p ≤ 0.05, **, p ≤ 0.01.

**Figure 3:**
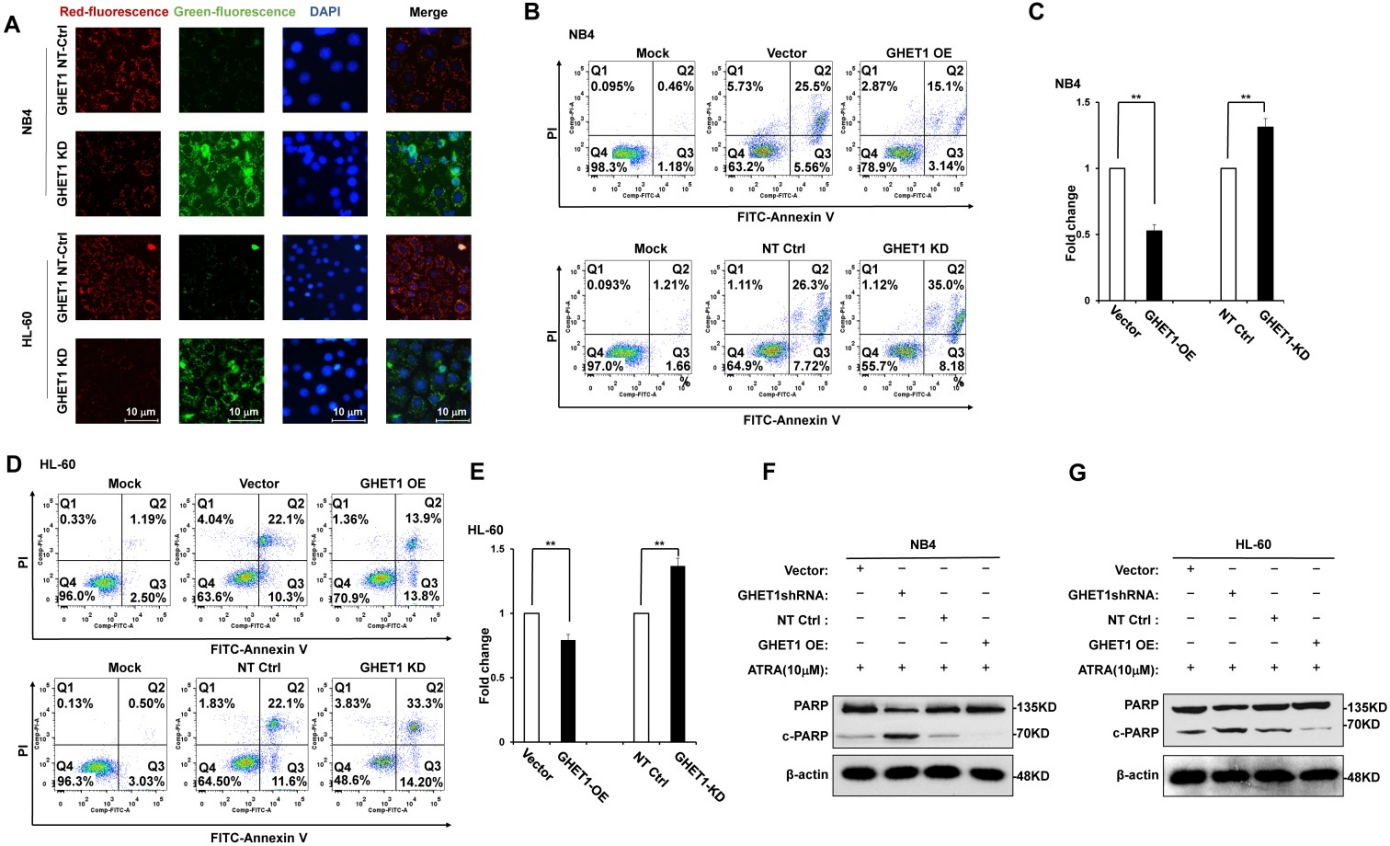
** Effects of GHET1 on cell apoptosis.** (**A**) Changes in the mitochondrial membrane potential in NB4 and HL-60 cells with GHET1 overexpression or knockdown were observed by JC-1 staining. (**B-E**) Cell apoptosis was detected by flow cytometry, and the fold change is shown in the histogram. (**F, G**) The protein expression of c-PARP was examined by Western blotting. GHET1 overexpression inhibited cell apoptosis, and GHET1 knockdown promoted cell apoptosis. *, p ≤ 0.05, **, p ≤ 0.01.

To detect the effects of GHET1 on the proliferation of AML cells, CCK-8 assays were performed. Cell proliferation was clearly suppressed after 12 and 48 h of infection with GHET1 shRNAs in HL-60 and NB4 cell lines, respectively (**Fig. 2A**). Cell proliferation was significantly enhanced after 12 and 48 h of transfection with GHET1 expression vector in HL-60 and NB4 cell lines, respectively (**Fig. 2B**). The effects of GHET1 on cell differentiation of AML cells were analyzed by flow cytometry. Compared with cells overexpressing GHET1, cells with GHET1 knockdown had an increased ratio of cells expressing CD11b in both cell lines (**Fig. 2C**). To further demonstrate the effect of GHET1 on AML cell differentiation, the expression levels of CD11b mRNA were analyzed by qPCR in AML cell lines with GHET1 overexpression or knockdown. The expression of CD11b was significantly decreased after GHET1 was overexpressed in AML cell lines compared with the control (**Fig. 2D**). Conversely, the expression of CD11b was significantly increased after GHET1 was knocked down in AML cell lines compared with the control (**Fig. 2E**), suggesting that GHET1 can inhibit cell differentiation. We also detected the effects of GHET1 on the apoptosis of AML cells by JC-1 staining and flow cytometry. Compared with control, the cell apoptosis rate had a significant increase or decrease after GHET1 was knocked down or overexpressed in NB4 and HL-60 cell lines (**Fig. 3A-E**). To ensure the confirmation of the influence of GHET1 on the apoptosis of AML cells, we verified expression of the apoptosis-related protein c-PARP by Western blot analysis. In both the NB4 and HL-60 cell lines, the protein expression of c-PARP was higher in the GHET1 shRNA group than in the GHET1 overexpression group and control group (**Fig. 3F, G**). These data showed that GHET1 could inhibit the apoptosis of AML cells, indicating that GHET1 may be an oncogenic gene in AML.

### GHET1 may directly target miR-105, and the GHET1/miR-105 axis contributes to the proliferation, differentiation, and apoptosis of AML cells

**Figure 4:**
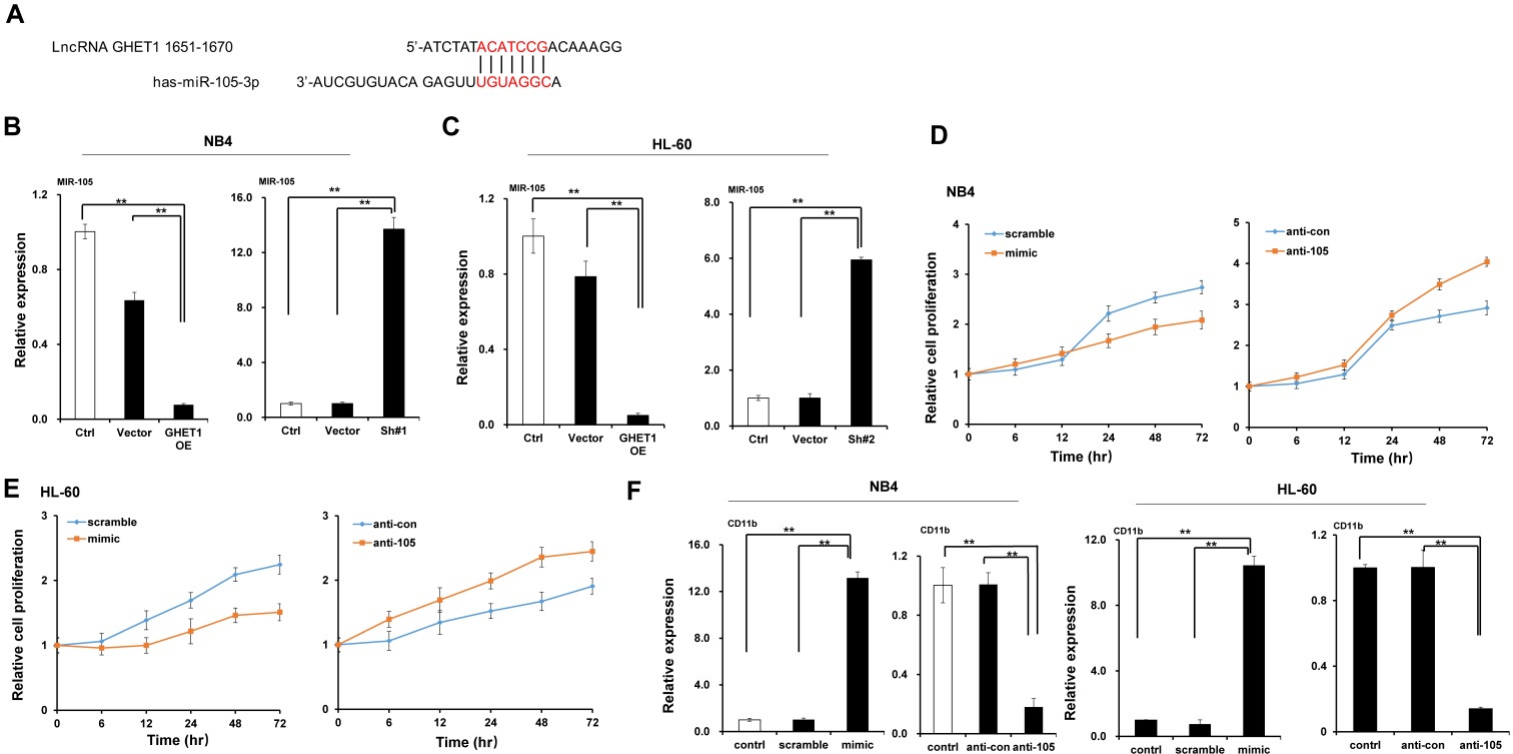
** GHET1 directly targeted miR-105, and the GHET1/miR-105 axis contributed to cell proliferation and differentiation of AML cell lines.** (**A**) Graphical presentation of 3′-UTR of GHET1 involving the suggested miR-105 target site. (**B, C**) The expression of miR-105 when GHET1 was overexpressed or knocked down was detected by qPCR. MiR-105 expression was markedly downregulated when GHET1 was overexpressed and markedly upregulated when GHET1 was knocked down. (**D, E**) Cell proliferation assay of NB4 and HL60 cells transfected with miR-105 mimic or miR-105 inhibitor using the CCK-8 assay. Reduced expression of miR-105 can promote cell proliferation, and increased expression of miR-105 can inhibit cell proliferation. (**F**) The CD11b expression in NB4 and HL60 cells with miR-105 overexpressed or inhibited was detected by qPCR. Reduced expression of miR-105 inhibited cell differentiation, and increased expression of miR-105 promoted cell differentiation. *, p ≤ 0.05, **, p ≤ 0.01.

**Figure 5:**
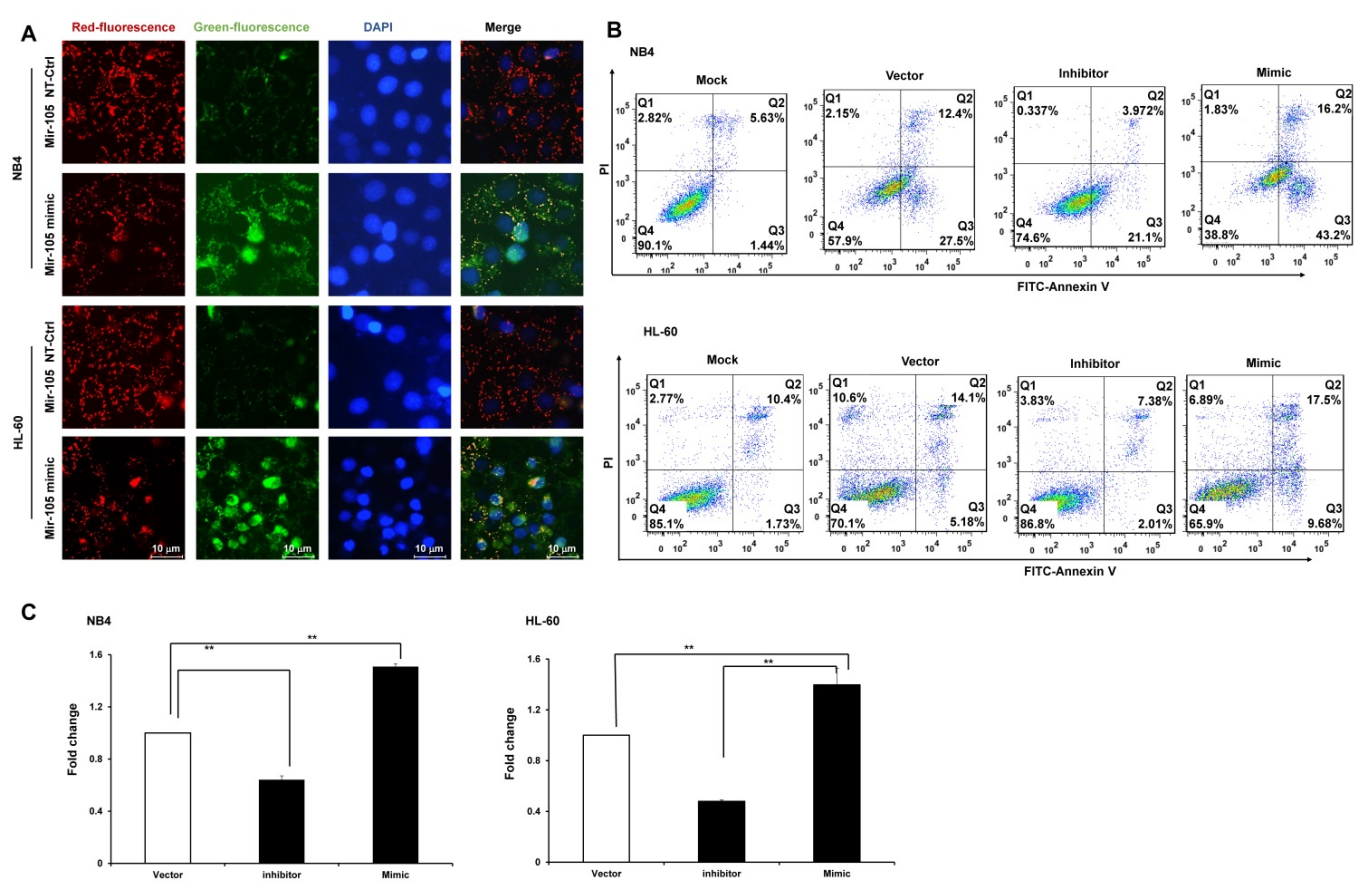
** GHET1/miR-105 axis contributed to cell apoptosis of AML.** (**A**) Changes in mitochondrial membrane potential in NB4 and HL-60 cells with miR-105 overexpressed or inhibited were observed by JC-1 staining. (**B, C**) Apoptosis of NB4 and HL-60 with miR-105 overexpressed or inhibited was detected by flow cytometry, and the fold change is shown in the histogram. Reduced expression of miR-105 inhibited cell apoptosis, and increased expression of miR-105 promoted cell apoptosis. *, p ≤ 0.05, **, p ≤ 0.01.

Bioinformatics predicted the miRNA target sites in GHET1, and miR-105 was studied in the subsequent experiments (**Fig. 4A**). We detected the expression of miR-105 in NB4 and HL-60 cell lines when GHET1 was overexpressed and knocked down. MiR-105 expression was markedly downregulated when GHET1 was overexpressed and markedly upregulated when GHET1 was knocked down compared with the control (**Fig. 4B, C**). The results demonstrate that GHET1 may negatively affect miR-05 expression. To determine whether the biological function of GHET1 in AML was regulated by miR-105, we overexpressed or suppressed the expression of miR-105. Cell proliferation in miR-105 mimic group was substantially decreased compared to the scramble group in AML cell lines, and cell proliferation in the anti-miR-105 group was substantially increased compared to the anti-control group in the AML cell lines (**Fig. 4D, E**). The expression of CD11b in the miR-105 mimic group was substantially increased compared to the scramble group and control group after transfection in the AML cell lines. Conversely, the expression of CD11b in anti-miR-105 group was substantially decreased compared to the anti-control group and control group after transfection in AML cell lines (**Fig. 4F**), suggesting that miR-105 can promote cell differentiation. We also evaluated the effects of miR-105 on cell apoptosis of AML cells by JC-1 staining and flow cytometry. Cell apoptosis in the miR-105 mimic groups were substantially increased compared to the nontarget control (NT-Ctrl) group in AML cell lines (**Fig. 5A**). Moreover, the cell apoptosis rate had an obvious increase after miR-105 mimic was transfected compared with the inhibitor group and the control, and cell apoptosis clearly decreased after miR-105 was inhibited compared with the mimic group and NT-Ctrl group in NB4 and HL-60 cell lines (**Fig. 5B, C**). These data showed that miR-105 could promote cell apoptosis. Together, these results suggest that GHET1 can directly target miR-105, and that the GHET1/miR-105 axis contributes to the proliferation, differentiation, and apoptosis of AML cells.

### MiR-105 directly targeted *RAP2B*

**Figure 6:**
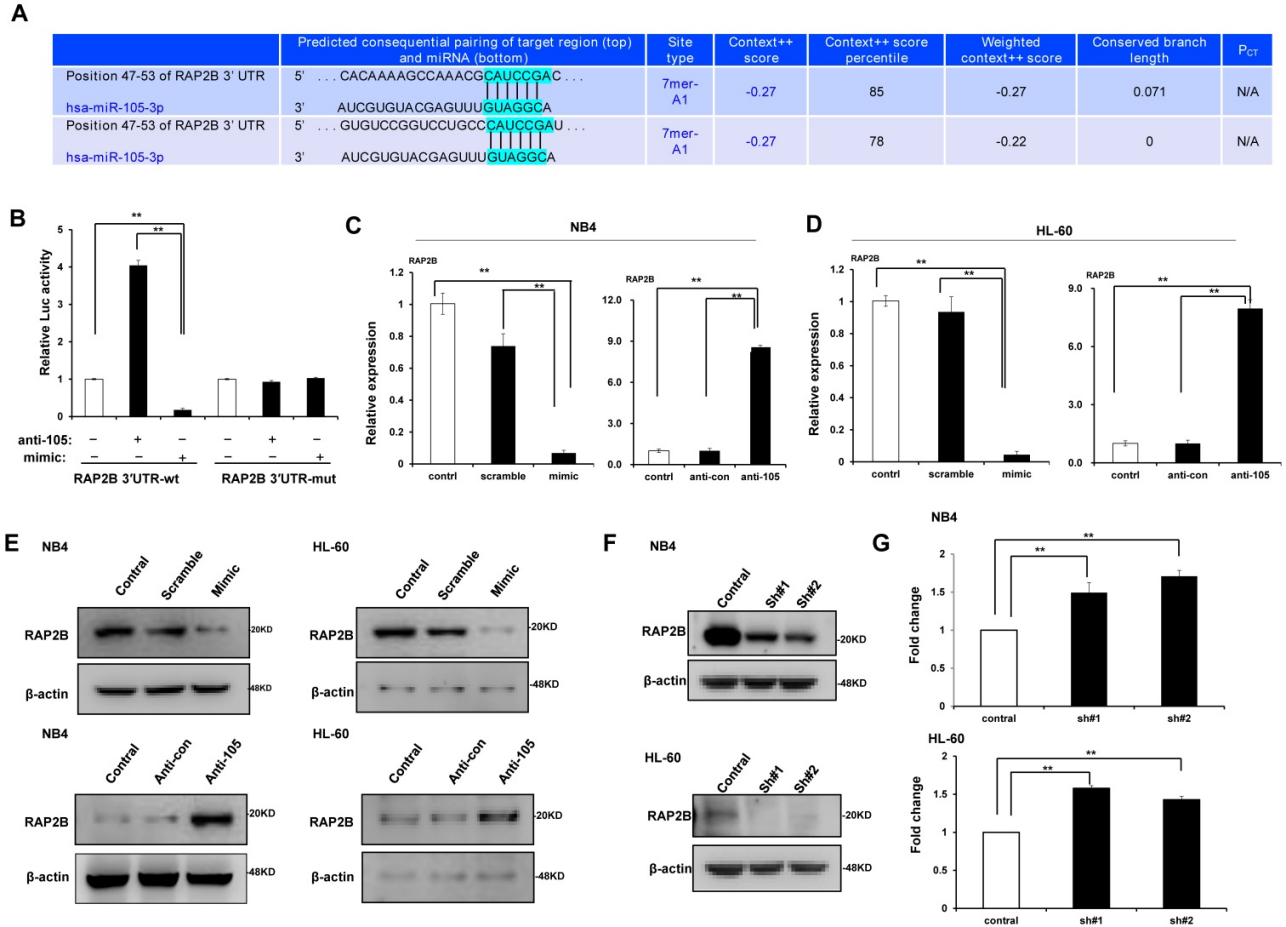
** MiR-105 overexpression decreased *RAP2B* level.** (**A**) Graphical presentation of 3′-UTR of miR-105 involving the suggested *RAP2B* target site. (**B**) The direct interaction between miR-105 and 3ʹ-UTR of *RAP2B* was detected by dual luciferase reporter assay. (**C, D**) The mRNA levels of *RAP2B* in miR-105 mimic groups were substantially lower than scramble groups and control groups after transfection, and those in anti-miR-105 groups were substantially higher than control groups and anti-control groups. (**E**)The protein levels of *RAP2B* in miR-105-mimic groups were substantially lower than control groups and scramble groups, and those in anti-miR-105 groups were substantially higher than control groups and anti-control *groups.* (**F**) The protein levels of *RAP2B* were substantially increased when cells were transfected *RAP2B* shRNA. (**G**) NB-4 cells and HL-60 cells were cultured in plates with 100 nm ATRA. After 24 h, cell apoptosis was analyzed with flow cytometry. Apoptosis of the *RAP2B* shRNA group was higher than that of the control group. *, *p* ≤ 0.05, **, *p* ≤ 0.01.

We predicted through bioinformatics that *RAP2B* is one target of miR-105. The alignment of miR-105 with 3ʹ-UTR of *RAP2B* is shown **Figure 6A**. Through the dual luciferase reporter assay, we demonstrated the immediate interaction between miR-105 and 3ʹ-UTR of *RAP2B*. Transfection of the miR-105 mimic reduced the luciferase activities of the 3′-UTR of *RAP2B*, but no impact on the luciferase activities by miR-105 mutant transfection (**Fig. 6B**). These outcomes showed that miR-105 targets 3ʹ-UTR of *RAP2B* to regulate expression directly. Moreover, miR-105 overexpression also affected the mRNA and protein levels of *RAP2B* upon transfection. The mRNA and protein levels of *RAP2B* in the miR-105 mimic groups were substantially decreased compared to the scramble and control groups after transfection, and those in the anti-miR-105 group were substantially increased compared to the control groups and anti-control groups (**Fig. 6C-E**). In addition, NB-4 cells and HL-60 cells transfected with *RAP2B* shRNA or empty plasmids (vector) were subjected to western blot analysis to detect levels of *RAP2B*. The level of *RAP2B* in NB4 cells and HL-60 cells was substantially decreased when cells were transfected with *RAP2B* shRNA (**Fig. 6F**). NB-4 cells and HL-60 cells were cultured in plates with 100 nm ATRA. After 24 h, cell apoptosis was analyzed by flow cytometry. The apoptosis of *RAP2B* ShRNA group was higher than that of the control group (**Fig. 6G**). Thus, *RAP2B* may serve as a functional target gene of miR-105 in human AML cells, and miR-105 may affect cell growth through *RAP2B.*

## Discussion

There is great heterogeneity in the etiology, pathogenesis, clinical characteristics, and efficacy of acute myeloid leukemia. In depth study of lncRNA may provide novel markers for the diagnosis of leukemia and new ideas and targets for the treatment of leukemia.

It has been verified that lncRNAs, as miRNA sponges or miRNA suppressors, interact with miRNAs and play key roles in human carcinogenesis by regulating the expression of miRNA target genes 21. In this study, we researched the roles of GHET1 in AML and the potential molecular mechanisms. First, we noticed that GHET1 expression was elevated in AML cells. To investigate the biological function of GHET1 in AML, cell proliferation, cell differentiation, and cell apoptosis *in vitro* by loss-of-function experiments and enhancement-of-function experiments were all measured. The data showed that GHET1 knockdown could suppress proliferation, and cause differentiation and apoptosis of AML cells *in vitro*, indicating that GHET1 works as an oncogene in AML.

Although it has been indicated that GHET1 works as an oncogene in AML, the potential mechanisms involved in tumorigenesis are unclear. We predicted that miR-105 can be a potential target of lncRNA GHET1 by bioinformatics analysis MIRDB4.0. Wang *et al*. 22 showed that lncRNA-linc00261 inhibited the proliferation and metastasis of non-small cell lung cancer by downregulating the expression of miR-105. Therefore, we assumed that the function of GHET1 in AML was also partially mediated though miRNAs. Thus, we examined miR-105 expression in AML cell lines. We demonstrated that the expression of miR-105 was greatly downregulated when GHET1 was overexpressed, and the expression of miR-105 was greatly upregulated when GHET1 was knocked down. Furthermore, we found that miR-105 overexpression could suppress proliferation, and induce differentiation and apoptosis of AML cells *in vitro*. There was consistency between our hypothesis and the experiment data, showing that GHET1 may work as an oncogene in AML by inhibiting miR-105 expression.

As a novel miRNA regulator, miR-105 plays a bidirectional regulatory role in the pathogenesis of tumors. MiR-105 acts as a tumor suppressor or an oncogene, depending on the particular tumor context and base-pairing genes. *RAP2B*, a member of the GTP-binding proteins, is generally increased in many types of tumors such as glioma 23, colorectal cancer 24, and renal cell carcinoma 19. We predicted that *RAP2B* may be a potential target of miR-105 by bioinformatics analysis (http://www.targetscan.org/). To determine whether GHET1-induced miR-105 suppression can cause an expression change in its underlying target genes, we concentrated on *RAP2B* for further studies. We proved the direct interaction between miR-105 and 3ʹ-UTR of *RAP2B* by the dual luciferase reporter assay. We also noticed that the expression of *RAP2B* would greatly increase when miR-105 was knocked down in AML cells. We also observed that the apoptosis of AML cells increased when *RAP2B* was knocked down. These results suggested that the GHET1/miR-105/*RAP2B* axis may be a significant signaling pathway involved in AML progression.

To summarize, we showed that high GHET1 expression participated in AML progression by promoting cell proliferation, and suppressing cell differentiation and apoptosis, which was mediated by inhibiting miR-105 expression and regulating *RAP2B* gene expression. These findings provide novel insights into the molecular mechanisms of the altered expression of lncRNA and miRNA in AML pathogenesis, and might facilitate the diagnosis and development of new treatments for AML patients.
